# Predictive factors of the general public’s willingness to be seen and seek treatment from a nurse practitioner in Australia: a cross-sectional national survey

**DOI:** 10.1186/s12960-021-00562-7

**Published:** 2021-02-17

**Authors:** Trudy Dwyer, Alison Craswell, Matthew Browne

**Affiliations:** 1 CQUniversity Australia, Building 18 Rockhampton Campus, Bruce Highway, Rockhampton, Q 4702 Australia; 2 School of Nursing, Midwifery and Paramedicine, University of the Sunshine Coast, 90 Sippy Downs Drive, Sippy Downs, Q 4556 Australia; 3 CQUniversity Australia, University Drive, Building 8/G.47, Branyan Australia, Bundaberg, Qld 4670 Australia

**Keywords:** Australia, National survey, Nurse practitioner, Consumer satisfaction, Consumer choice, Consumer experience

## Abstract

**Background:**

Health care delivery in Australia is experiencing challenges with services struggling to keep up with the increasing demands of an aging population, rising levels of chronic disease and limited funding for care. Where adjunct models of health care such as the Nurse Practitioner (NP) have the potential to address this gap, in Australia, they remain an underutilised service. Clarifying the nature of the consumers ‘willingness’ to be seen by NPs warrants further investigation.

**Methods:**

Australia-wide, cross-sectional population-based survey was undertaken using computer-assisted telephone interviewing technique.

**Results:**

While just over 53% of the general public participants (*n* = 1318) had heard of an NP, once they became aware of their scope of practice, the majority agreed or strongly agreed they were willing to be seen by an NP in the community (91.6%), the emergency department 88.2%), to manage chronic conditions (86%), to have scrips written and referrals made (85.3%), and if they did not have to wait so long to see a medical doctor (81%). Factors significantly predicting willingness were being: female, less than 65 years of age, native English speakers, or residents from town/regional and rural settings.

**Conclusion:**

Despite limited awareness of the NP role, a large proportion of the Australian population, across different demographic groups, are willing to be seen and treated by an NP. Expansion of this role to support medical services in areas of need could improve healthcare delivery.

## Background

Australia, like other countries in the developed world, is experiencing increased demand on the health care system. This demand is characterized by higher numbers of patient presentations and congestion in emergency departments, and long wait times to see the medical doctor/practitioner (MD) [1, 2]. Factors contributing to this issue are the increased proportion of the population who are older, have chronic or multiple health problems and who experience difficulty accessing timely or cost-effective primary health care such as general medical practitioners (GP) [2–7]. It is anticipated that this increasing demand for access to an already stretched primary health care services will not ease soon [8]. Internationally, health workforce shortages necessitate the implementation of multiple and targeted approaches involving a range of health workforce delivery models to provide support for those with chronic health conditions and co-morbidities, which in turn improves efficiency, reduces hospital admissions and improves the quality of life of the individual [8, 10, 11].

Internationally, one health professional group experiencing rapid role expansion is the Nurse Practitioners (NP). The NP is a registered nurse with additional qualifications, authorised to integrate advanced nursing and medical clinical skills, to independently assess, diagnose, prescribe medications, and manage patients within an agreed scope of practice [9]. While the title protection, regulation, qualifications and scope of practice of the NP role differs across countries [9], in Australia, to be endorsed as an NP, the registered nurse candidate must hold a Master’s degree and 5000 h or 3 year full-time specialised clinical experience [12–17]. The scope of the Australian NP practice includes: managing patient episodes of care, initiation and interpretation of diagnostic and pathology investigations, endorsement to prescribe medications, patient education and health promotion, admission and discharge rights and referrals for specific patient groups [10, 11, 18–21]. As increasingly more NPs are getting established in the Australian public sector workforce [17, 22] and to a lesser extent in the private, community and primary care settings [12], the question as to what patients want continues to emerge [23].

Internationally and in Australia, health service providers are reconceptualising how health care is delivered through adjunct, autonomous models of care, delivered by health professionals who work independently to manage caseloads and who are not a medical doctor [9, 14, 21, 24]. Patient reported outcome measures such as patient experience and satisfaction are fundamental measure of quality health care [25]. Patient perception of care is important as satisfaction correlates with compliance, improved health outcomes and quality of life [23]. While consumer experience and satisfaction with the NP role is well documented in the international and Australian literature [9, 11, 26–36], much of this evidence has been collected during or following NP consultations. Evidence is needed understanding consumer choice and public expectations or willingness to see different health practitioners. Indeed, authors caution that health care providers should not assume that all people are willing to receive autonomous health care from personnel such as NPs who are not an MD [37, 38].

The emerging international and Australian evidence is that the general public are willing to treated by health professionals who are not an MD in circumstances, where they would have traditionally only consulted the MD [39–41]. Circumstances included; minor ailments [37] reduced waiting time for a consultation [37, 42]; and the extra time they receive from nurses [31, 34, 43–46]. Evidence from the U.S shows that once having treated solely by the NP, instead of an MD, 94% patients reported a willingness to be seen by the NP on future visits [43]. Clarifying the nature of the consumers ‘willingness’ to be seen by health care workers warrants further investigation [38, 40, 42, 43, 47].

Australian studies have been undertaken to ascertain health-care consumers’ willingness to be seen by an NP [40] and what level of independent treatment would they accept from the NP for their primary health care needs [22, 48]. Parker et.al reported that Australian health consumers are supportive of the NP role to provide medical certificates, repeat prescriptions, and treat ‘minor’ or ‘every day’ health concerns [22, 48]. A limitation of Parker and colleagues’ [22, 48] research is that the participants reported either limited or no prior experience of seeing seeking and receiving treatment from a health professional who is not an MD. Participants experienced confusion around role delineation and differences between nurse practitioners, other nurses and GPs [48].

In Australia, the NP role has only been in place for a relatively short period, achieving legislated title protection in 1998 [19] with current estimate of 1745 endorsed practitioners nationally [17]. Given this nascence, it is fair to assume that the public are not familiar with NP role and scope of practice. With the exception of Parker and colleagues’ [22, 48] research, there is a paucity of empirical data focused on public willingness to be seen and treated by an NP. Exploring the general public’s willingness to engage with an unfamiliar model of health care can potentially have important implications for service delivery [34]. The aim of this study was to contribute new knowledge in this field by examining the factors related to the general public’s willingness to be seen and treated by an NP.

## Methods

### Design

This population-based cross-sectional survey of Australian residents sought to identify factors that predict the general public’s willingness to be seen and seek treatment from an NP. Secondary aims were to determine:

- the proportion of the Australian population willing to be seen and treated by an NP,
- if socio-demographic factors (i.e., age, sex, education, location) of individual’s willingness to be seen by an NP
- if waiting time reduction influences willingness to be seen and treated by an NP.

### Sample

The study was designed to create an estimation of the attitudes of Australian adults, 18 years and over, on the topic of interest [49, 51]. The research team describes this technique in detail elsewhere [50, 52]. For sampling purposes, Australia was stratified into state and territory areas and telephone numbers randomly selected using random digit dialing (RDD) databases supplied by Sampleworx Pty Ltd. Approximately 48% of the sample were contacted on a mobile telephone. To ensure equal representation of males and females, the sex of the potential participant was randomly selected prior to making the phone call. To be eligible, the participant has to be over the age of 18 years and be of the predetermined sex.

We used the most recent total population according to the Australian Bureau of Statistics August 2015 for 18 + which was 18,182,764. The sample size of 1318 was considered to be large enough to yield suitably precise estimates of prevalence rates of interest, with 95% confidence intervals of approximately ± 1.5%.

### Survey measures

The survey questions were developed from literature and existing survey [26, 48].

All data were collected by a research assistant, who read the questions to the participant over the telephone. Both the survey questions and data collector technique were pilot-tested (Table 1) with 40 randomly selected dwellings. Minor question and data collector script changes were made to increase validity by addressing inadequate responses and changing confusing wording. The survey consisted of a standardised introduction, demographic and core general health questions, seven closed-ended (Likert scale) questions specifically elicited information about willingness to see NP (Table 1) and two open-ended questions. Subsequent to participant consent to participate, the data collector asked if the person had heard of an NP, then they read out a description of the role of the NP (as outlined in Table 1) prior to proceeding. This description of the NP served to ensure all participants had same understanding of the role of the NP. During data collection, 10% of the data collectors were monitored for consistency.

**Table 1:** Closed-ended survey questions about NP attitudes

Have you ever heard of a Nurse Practitioner?
*Given that NP role is new within Australia the interviewer then clarified the role of the NP for the participants by reading out*
“Nurse practitioners are registered nurses with a Master's degree and extensive clinical experience. They can refer patients to other health professionals, prescribe medications such as antibiotics and order diagnostic tests such as X-rays or blood tests. They work independently, or as part of the health care team to treat conditions that are traditionally provided by the doctor.”
*Prior to asking of each of the NP opinion questions the interviewer read out the following statement;*
“Bearing in mind that I would still be referred to a doctor if necessary, I would be willing to receive care from a Nurse Practitioner”:
If the Nurse Practitioner spent more time with me than I usually receive when I attend the Doctor.
If the Nurse Practitioner could treat my child.
If I didn't have to wait so long to see the doctor.
If the Nurse Practitioner were able to write prescriptions, order pathology, x-rays and refer to Specialists.
If the Nurse Practitioner were able to write prescriptions, order pathology, x-rays and refer to Specialists.
If the Nurse Practitioner could manage my chronic or continuing condition.
In the Emergency Department.
In the Community Setting (for example GP, community health).
If you were given the option of seeing a Nurse Practitioner immediately, OR waiting for a period of time to see a Doctor, how long would you be prepared to wait to see to doctor?

Demographic variables included age, education, locality and sex. Multiple general health variables were also included; the number of days in the last month that health interfered with daily activities, days in poor physical health, days in poor mental health, and days in which sleep was inadequate. Health status responses were nominally coded; 0 days (0), 1–10 days (1), 11–20 days (2), and 21–30 days (3). Also included as predictors were general health ratings; poor (0), fair (1), good (2), and whether the individual was suffering from a chronic condition. The response variables that were not intrinsically binary were transformed as necessary for data analysis.

The interviewer also asked two open-ended questions “Could you briefly describe a situation and why you would /would not consider using a nurse practitioner?” Given the volume of the public responses to this qualitative component of the study, the findings are presented elsewhere [50]. This paper reports the quantitative responses from the survey.

### Data analysis

NP attitude responses were measured using a five-point Likert scale and collapsed into two discrete categories; agree (agree/strongly agree) or fail to agree (neutral, disagree, strongly disagree) prior to analysis. On any given item, relatively few (1.8%) respondents either did not know the answer or made no response. These non-responses were treated as missing data and excluded from analyses on a variablewise basis. Descriptive statistics were used to describe demographic profiles and Wilcoxon rank sum test used to determine differences in willingness scores. We used R statistical package and conducted a series of exploratory logistic regressions to isolate significant demographic and health status predictors of the general public’s willingness to see an NP. Stepwise backwards variable selection was performed, optimising the Akaike Information Criteria (AIC). This is a model selection technique that attempts to minimise the deviance (error) whilst maximising model parsimony (fewer predictors) [54]. Associations are presented as an odds ratio (OR) in comparison with a reference group and indicated the increased or decreased likelihood of a sub-group within the population to perform a specific behaviour. Statistical significance was set at < 0.05.

## Results

Of those eligible, 1318 (response rate 33%) residents agreed to participate in the telephone survey, representing all Australian states and territories (Table 2) and slightly more females (54.3%, *n* = 716) than males (45.7%, *n* = 602). Reasons people declined to participate included, a reluctance to participate in telephone surveys; screening of calls; and the anticipated length of the survey (average total length of telephone survey = 33 min). Most were born in Australia (75%, *n* = 989), in married/de-facto relationships (66.5%; *n* = 877), employed on full/part-time basis (52.9%; *n* = 697) and held a technical or higher education qualification (66.4%; *n* = 875). Just under a third were retired or on a pension (29.2%; *n* = 385). The sample had a mean age of 52.6 years (SD 17.96) and range of 18–101 years.

**Table 2:** Demographic profile (*n* = 1318)

Item	*n* % of total sample		% of sample heard of NP	*P* value
Total population			53.3	
Sex				
Male	602	45.7	43.9	.001
Female	716	54.3	61.3	
Age				
18–34 years	255	19.3	43.9	.003
35–44 years	186	14.1	48.4	
45–54 years	219	16.6	53.0	
55 years and over	645	48.9	58.8	
Highest level of education				
Primary schooling or below	26	1.9	48.3	.001
Secondary/High School	410	31.1	46.3	
Technical studies or further education	292	22.2	45.9	
University or Tertiary	583	44.2	62.4	
Australian State or Territory				
Australian Capital Territory	33	2.5	54.5	.845
New South Wales	380	28.8	52.9	
Northern Territory	28	2.1	57.1	
Queensland	271	20.6	50.6	
South Australia	89	6.8	52.8	
Tasmania	35	2.7	71.4	
Victoria	347	26.3	53.6	
Western Australia	131	9.9	53.4	
Rurality				
City	652	49.5	54.0	.924
Town/regional	321	24.4	51.7	
Rural	345	26.2	53.7	

### Heard of nurse practitioner

Overall, just over half (53.3%; *n* = 703) of respondents had previously heard of an NP with 4% (*n* = 46) were unsure whether they had or not. Within this group, two-thirds (61%; *n* = 439) of female respondents had heard of an NP compared with less than half of the participating males (44%; *n* = 264) (*χ*^2^ = 41.07, *df* = 1, *p* < 001). Around two-thirds of people over the age of 55 years (58.8%; *n* = 379) and those with a tertiary education (62.4%; *n* = 364) were also significantly more likely to have heard of a nurse practitioner (Table 2).

### Willingness to receive care from the nurse practitioner

**Fig. 1:**
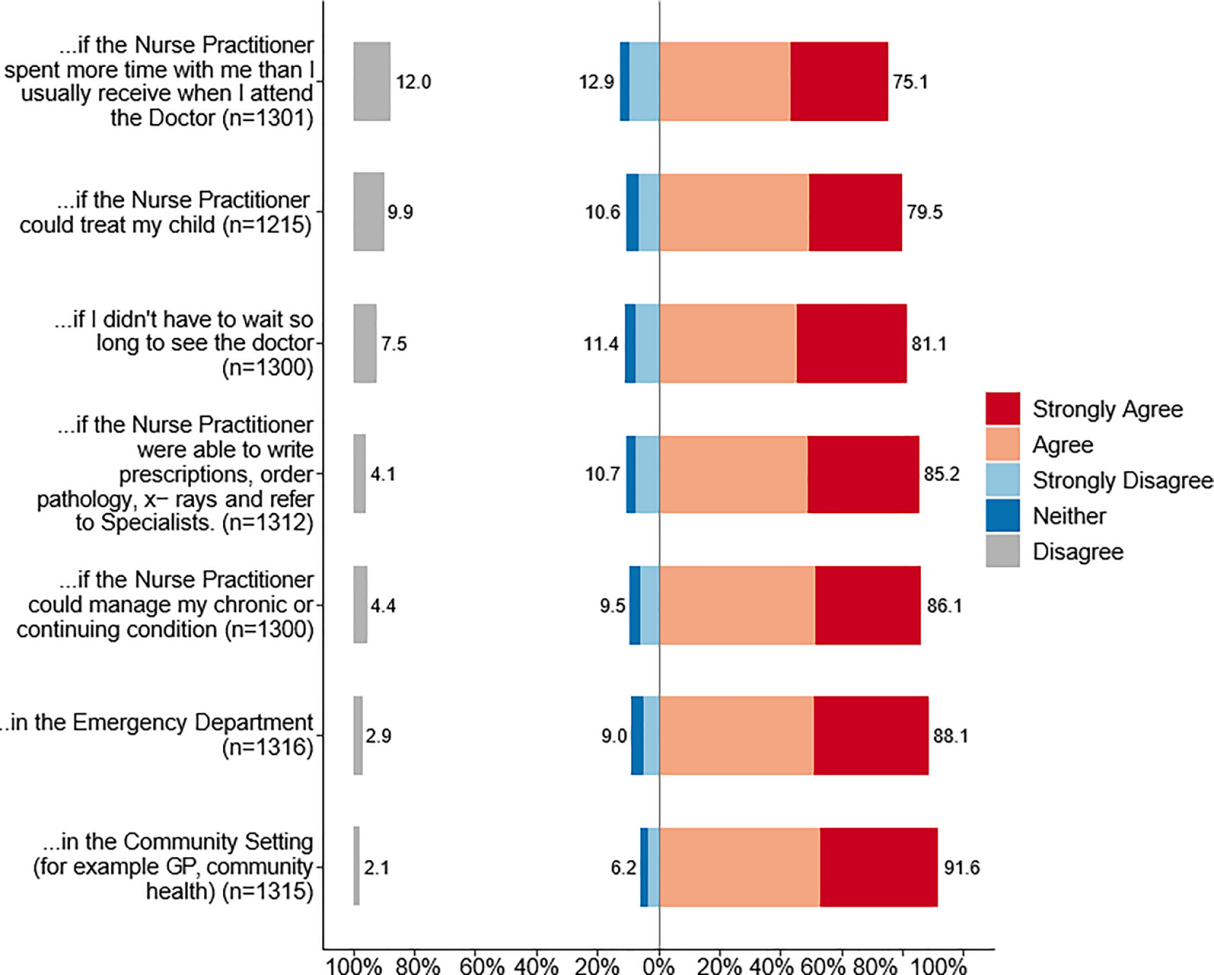
Percentage of positive and negative responses to items ‘I would be willing to receive care from a Nurse Practitioner…’

The majority of the respondents agreed/strongly agreed that they were willing to be seen by the NP for a variety of health care scenarios. A total of 91.6% (n = 1205) of respondents were willing to see an NP in a community treatment setting, whilst 75.1% (*n* = 977) responded positively on the condition that the NP would spent more time with them than a doctor. Figure 1 compares variation in responses regarding willingness to receive care from an NP in various circumstances.

As each of these questions (Fig. 1) concerned willingness to utilise an NP, we considered whether it would be valid to aggregate the items in a single scale. The polychoric inter-correlations of these items ranged between 0.60 and 0.80, and the Mokken scalability coefficient *H* for the entire scale was 0.64, well above the recommended threshold of 0.30 [53, 54]. Accordingly, we calculated a mean ‘willingness to receive care from an NP’ score for each participant from the seven items (Fig. 1). There was no relationship between having previously heard of an NP and willingness to receive care, both those who had (M = 4.07, SD.83) and those who had not (*M* = 4.05, SD 0.775) had a mean response close to ‘Agree’ (Wilcoxon rank sum test *W* = 195,250, *p* = 0.461). Older respondents tended to be less willing to see an NP (Spearman *r* = − 0.15). While not statistically significant (*p* = 0.064) women (*M* = 4.11; SD 0.80) were slightly more willing to see an NP than men (*M* = 4.02, SD.79).

### Reduced wait time

**Fig. 2:**
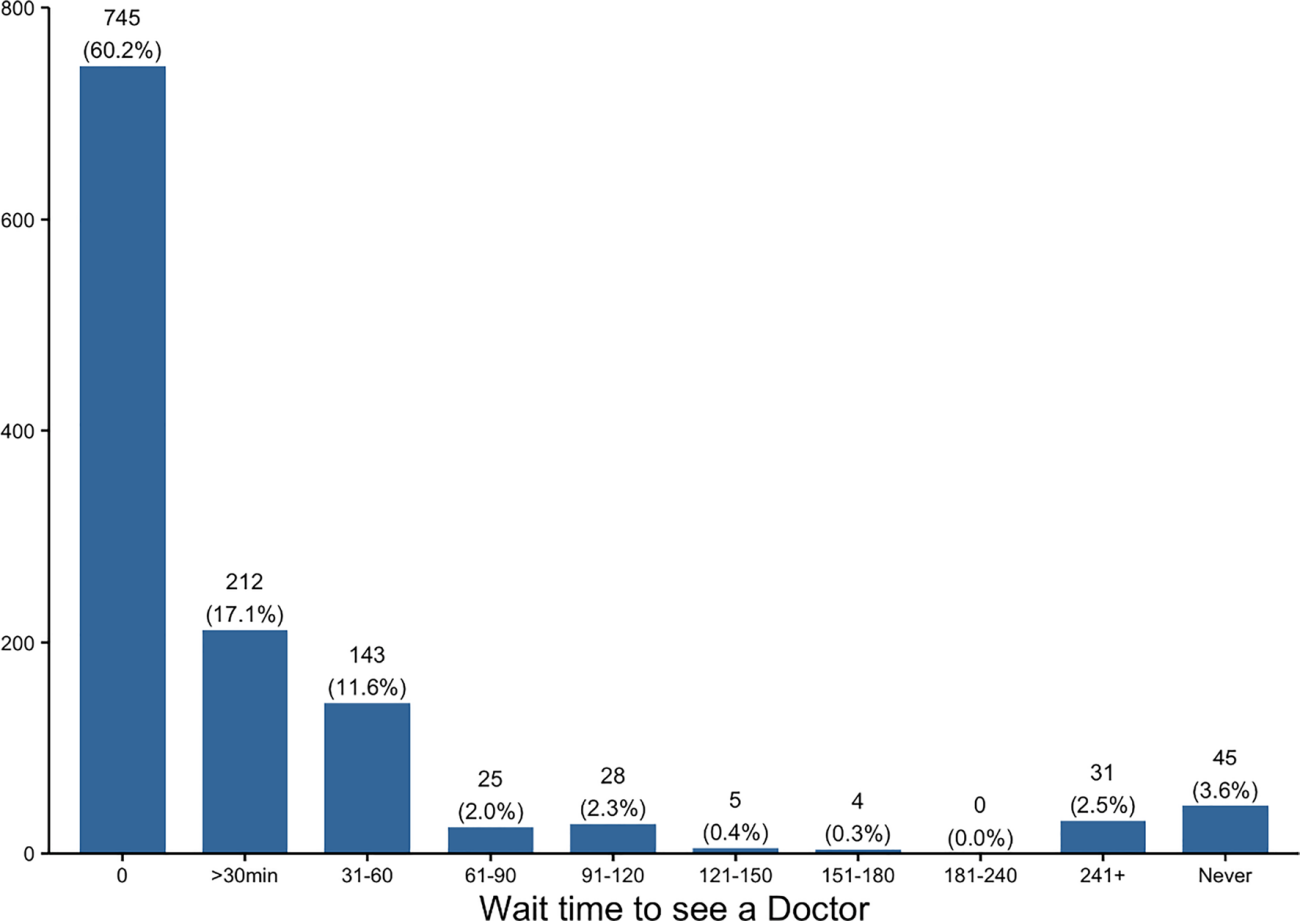
Duration of time willing to wait to see an MD in preference to an NP. Zero (0) indicates preference to see an NP immediately

The majority (60.2%, n = 745) of respondents preferred to see an NP immediately rather than having to wait to see the MD. A small proportion (2.5%, *n* = 31) of participants would prefer to wait over 4 h to be seen by an MD or would not see an NP under any circumstances (3.6%, *N* = 45) (see Fig. 2). 640 participants (48.6%) affirmed that there were situations in which they would not consider an NP. Yet, another 641 respondents (48.6%) indicated that they would always consider an NP and 6% did not respond or were unsure.

In the interest of understanding whether the personal factors that might affect attitudes to being seen by an NP, we conducted a series of exploratory logistic regressions on each of the NP-willingness related items, predicting (Agree [1] versus Disagree/Neutral [0]. These categories were combined due to their relatively low prevalence. Factors that best predict willingness to be seen by the NP are presented in Table 3. When controlling for these variables, the logistic regressions demonstrated that those over the age of 65 years (older respondents), when compared to all other age groups, were more likely to have heard of NPs, they were also less willing to receive care from an NP regardless of the service, even if they could see the NP sooner (Table 3, row 3). This older group of respondents were also more willing to wait longer periods of time to see a doctor. Interestingly, older respondents were also the cohort most likely to always consider an NP. In contrast, respondents from rural areas, when compared to people from cities, are more likely to always consider NP for prescriptions and referrals and to manage their chronic illness (Table 3, columns 4, 6). They were also less likely to want to wait to receive care from the MD. There were no observed differences in willingness to receive care from the NP between respondents who identified as having poorer physical health, mental health or a chronic disease compared to those who did not identify with the having these lower general health variables (Table 3, rows 12, 13).

**Table 3:** Factors contributing to respondents willingness to receive care from an NP in different circumstances

	Response variable									
	Heard of NP	Willingness to receive car from NP^a							Prefer Wait Dr^b	Always Consider NP
		ED	Community	Prescribe refer	More time	Manage chronic	Treat child	Less waiting		
(Intercept)	− 1.43*** (.22)	2.34*** (.23)	3.14*** (.36)	2.15*** (.30)	1.03*** (.25)	1.33*** (.29)	1.64*** (.18)	2.16*** (.21)	− 0.40* (.16)	− 1.14*** (.13)
Gender										
Female	–	–	–	–	–	–	–	–	–	–
Male	− 0.71*** (.12)				− 0.28* (.13)	− 0.25 (.16)				
Age (65+)	0.52*** (.14)		− 0.72*** (.21)	− 0.68*** (.29)	− 0.63*** (.15)	− 0.28 (.19)	− 0.77*** (.17)	− 0.69*** (.17)	0.45** (.14)	0.46* (.18)
Education										
Secondary	–	–	–	–	–	–	–	–	–	–
TAFE	0.10 (.16)	− 0.40 (.25)			− 0.30 (.19)		− 0.04 (.21)	− 0.21 (.21)	− 0.35* (.17)	
Tertiary	0.88*** (.14)	− 0.53* (.22)			− 0.61*** (.16)		− 0.36* (.18)	− 0.65*** (.18)	− 0.02 (.14)	
Days poor physical health	0.1 (.06)	− 0.17 (.11)		0.18 (.11)						− 0.13 (.08)
LOTE	− 0.46* (.18)	− 0.49* (.23)	− 0.45 (.28)	− 0.39 (.23)		− 0.71*** (.21)	− 0.55** (.20)	− 0.41* (.21)	0.40* (.18)	0.39 (.23)
Days poor sleep		0.19* (.09)		0.24** (.08)	0.17* (.07)	0.12 (.08)	0.19** (07)	0.13 (.07)		
Days health interfere		0.38** (.15)		− 0.28* (.12)						
Chronic disease		− 0.3 (.19)						− 0.35* (.16)	0.19 (.13)	
Location										
City	–	–	–	–	–	–	–	–	–	–
Town			0.46 (.26)	0.20 (.20)		0.30 (.20)			− 0.15 (.15)	− 0.29 (.20)
Rural			0.46 (.26)	0.44* (.21)		0.47* (.21)			− 0.55*** (.15)	− 0.56** (.20)
General health rating			− 0.31* (.15)	(.12)						
Days poor mental health									− 0.06 (.06)	
AIC	1629.82	927.92	738.19	1048.94	1401.75	1023.3	1177.98	1203.18	1614.95	993.43
Num. obs.	1248	1291	1290	1287	1276	1277	1196	1276	1219	944

^***^p < 0.001, ^**^p < 0.01, ^*^p < 0.05

^a Likert response transformed to binary Agree (1) vs Disagree / Neither (0)

^b Respondents willing to wait (any amount of time) to see a Doctor rather in preference to NP

## Discussion

This study provides confirmation of the general publics’ willingness to be seen and treated by an NP. When provided with information about NPs scope of practice, there was strong overall public support for this role across different demographic groups. Despite limited awareness of the role, the proportion of the Australian population in this study willing to be seen and treated by an NP was high. The majority reported high levels of willingness to be seen and treated by an NP (80–91.5%) in the community, the emergency department, to manage chronic conditions, to have scrips written and referrals made, and if they did not have to wait so long to see a medical doctor (MD). The main factors that predict greater willingness to be seen and receive treatment from an NP were being: female, under 65 years of age, a native English speaker, and a rural resident. Offering individuals immediate assessment by an NP as opposed for waiting for an MD review did improve the willingness to be seen and treated by an NP; however, this cannot be applied to all medical contexts.

This study indicates that the Australian public have limited awareness of the NP role with only half of the sample having heard of an NP, a finding consistent in contemporary literature [22, 48]. This is not surprising given the general publics’ limited understanding of the different categories nurses or levels within nursing groups [26, 49], the relatively low number of NPs (approximately 0.5% of the nursing workforce [14, 55]) and the fact that the majority of NPs in Australia are predominantly employed in the public sector [1]. International cross-country comparisons suggest that public exposure to the NP role will also be influenced by the fact that there are only 4.4 NPs per 100,000 population compared with 395 physicians per 100,00 population [14]. While the number of NPs nationally and internationally is slowly increasing [11, 14, 56], there is a risk that the general public, in failing to recognise the extended scope of the NP role in comparison to other nurses, nor their ability to independently manage a case load, may fail to recognise the NP role as a skilled and viable model for transforming health care delivery across Australia. In our study, we found that while there is limited understanding of the NP role amongst the Australian public, this had no relationship to their willingness to receive treatment and autonomous episodes of care from an NP. Indeed, a recent international scoping review of patient satisfaction with independent care provided by adjunct health care providers, such as the physician assistants, found that people are generally satisfied with care received, regardless of medical provider [23]. Health care consumer support, willingness and acceptance for autonomous and complementary models of health care are fundamental to informing policy change [24]. Our study affirms that the Australian public are very accepting of the NP model of care. This high level of acceptance is potentially related to the general public’s confidence in the governance of health the system to adequately educate the NP for the role [50].

In Australia, few NPs are employed in primary care and community settings [12, 17], and this fact provides context as to why only half of the participants had heard of the NP. Regardless of this limited awareness, no differences observed between respondents with a prior experience of accessing consultations with NPs and those without. Indeed, nearly, all (91.6%) of the participants in our study were willing to see an NP in a community setting. Our findings suggest that Australian public are open to independent nursing led models to access community and primary health care services and this was more evident in the younger population (less than 65 years of age). We also found that willingness to access the NP in the community setting (91.5%) and to manage a chronic illness (88%) were both very high. Maier et al. [14] proposed that 67–93% of primary health care services and visits can be delivered safely by an NP. This observation contrasts with Parker et al. study [22] who found that the management of chronic or long-term conditions was considered only moderately acceptable within the scope of an NP. A possible explanation for those over the age of 65 (when compared to all other age groups) being less willing to see an NP may well be related to the stero-typical understandings held by this age group and the legacy of the subservient role of the nurse to the MD [24]. Alternative explanations may be the Australian health-care consumers’ inexperience with the NP as an independent consultant for primary health care concerns and availability of and access to health care.

A cited feature of NP practice is longer consultations, of around 1 h in duration, allowing time for patients and their families to feel heard and listened to [31]. Arguably, these longer consultations have influenced patient satisfaction, their ability to understand, manage and cope with their illness [31, 45, 46]. Patient enablement has been attributed to the processes within the NP consultation, such as: the use of longer consultation times, the building of partnerships between NPs and patients, and through NPs’ holistic and hands-on consultation approach. Other studies report the longer consultation times improve patient adherence to treatment plans, positively impact changes in health behaviours [31] and improved service delivery [57]. Despite these espoused correlations between extended consultation times and reported patient satisfaction, we found that spending more time during consultations was not a contributing factor to willingness to be see an NP. A recent study found that NPs do not have lengthy consultation times, rather they convey a sense that they are spending more time during consultations [55]. Indeed, no correlation was found between the length of NP consultations and post-consultation consumer satisfaction or enablement [55]. These authors concluded, it is the consumers’ expectations and extent the NPs can make autonomous diagnostic and prescribing decisions that positively impact postconsultation enablement.

Timely access to health care is important, with 89% of those surveyed stating they were willing to be seen by the NP immediately rather than having to wait for over an hour to be seen by the medical doctor (MD). Similar findings were reported by Parker and colleagues [48]. Furthermore, in our study, people from regional towns and rural locations were significantly more likely to prefer to see the NP immediately as opposed to having to wait for the MD. This observation may reflect the difficulties people from these regional and rural settings experience accessing timely health care. Shortages of skilled health care workers, particularly specialist physicians, are widespread in rural and remote communities [14]. The geographical imbalances of access to health care means rural residents must either wait or travel some distances to access specialist services. NPs are well positioned to address this deficit and fill the service gap [58]. One part of the impetus for establishing NP roles in Australia was to increase patient access to care specifically in rural and remote settings [10].

In 2010 in Australia, the legal scope of the Australian NP practice was expanded to include ability to register consumers to access the Medicare Benefit Schedule (MBS) and the Pharmaceutical Benefits Scheme (PBS) [11]. These schemes ensure the NP can receive remuneration for specific services on the Schedule list [10, 57]. While NPs eligibly for MBS and PBS have facilitated the growth of independent (private) NP services the, restrictive nature of the MBS items is counterproductive, limiting the level of remuneration available, increasing the cost of NP service, and ultimately the general public’s acceptance of and utilisation of NP services [12, 17, 59, 60]. The design of the MBS and PBS and the mandate that NPs must establish collaborative arrangement with an MD or an establishment that employs MDs [17] are significant barriers to NP providing complete episodes of care, reducing their capacity to function autonomously [12]. Reviewing of or removing these mandated collaborative arrangements and extending access to MBS/PBS items will ensure NPs are able to function at their full capacity and thus improving access to care to reach underserved populations [7, 17]. The general public are accepting of the NP model of care as a means for accessing autonomous, safe and efficient health care, particularly when there are gaps in existing health services such as primary health care and in smaller towns or rural settings.

### Limitations

All survey research is limited by the nature of being self-reported. Of those who passively declined participation in the study, many may not have answered their phone due to screening of spam telephone calls or numbers that are unknown. Some may not have had mobile phone reception coverage or may not have been home at the time of the landline call. This sampling bias was accounted and corrected for with the use of data weighting of the results. Survey questions designed to be short and concise may have limited the respondent’s understanding of what was being asked and impact the quality of their response. In addition, the anonymity of participation in telephone surveys may bias outcomes [61]. Finally, providing the respondent with information about the NP role during the survey may have inadvertently contributed to informing their response.

## Conclusions

We found that the general public are overwhelmingly accepting of the NP model of care as means of increasing access to health services. Furthermore, the persons level of acceptance varies depending on their personal health circumstances, age and health status. This was more evident for people from smaller towns and rural settings, if accessing the NP services meant accessing care sooner. Given the NP role was introduced to fill gaps in health service and increase access to health care services for underserviced populations such as rural settings, it is now time to cease the rhetoric and support this highly skilled workforce to practise autonomously to meet public expectations.

## Data Availability

The datasets used and/or analysed during the current study are available from the corresponding author on reasonable request.

## Abbreviations

MD: Medical Doctor, broad term including general practitioners, physicians and hospital doctors
NP: Nurse Practitioner
